# A Manual of Human Anatomy

**Published:** 1853-04

**Authors:** 


*A Manual of Human Anatomy—Descriptive, Practical, and General.* By Robert Knox, M. D., F. R. S. E., Lecturer on Anatomy, and Corresponding Member of the National Academy of France, pp. 672, 12mo. London. Henry Renshaw, 1853.

This work is illustrated by 250 wood engravings of high finish, and can be obtained of Lindsay & Blakiston, book publishers, Philadelphia.

This Manual of Dr. Knox, who has long been a teacher of anatomy, and who knows full well the wants of the medical student, is, in our opinion, second to no similar work.

